# Vaginal Natural Orifice Transluminal Endoscopic Surgery (vNOTES) in Patients with Gynecological Malignancies: A Systematic Review

**DOI:** 10.3390/jcm15114089

**Published:** 2026-05-25

**Authors:** Aristotelis-Marios Koulakmanidis, Evangelia Kontogeorgi, Dimitrios Zacharakis, Anastasia Prodromidou, Ioakeim Sapantzoglou, Giuseppe Mascellino, Konstantinos Kypriotis, Nikolaos Kathopoulis, Dimos Sioutis, Charalampos Voros, Christos Vrysis, Stavros Athanasiou, Themos Grigoriadis

**Affiliations:** 1 1st Department of Obstetrics and Gynaecology, Alexandra Hospital, National and Kapodistrian University of Athens, 115 28 Athens, Greece; evangelia-kont@hotmail.com (E.K.); dimzac@hotmail.com (D.Z.); a.prodromidou@hotmail.com (A.P.); kimsap1990@gmail.com (I.S.); kypriotiskonstantinos@yahoo.it (K.K.); nickatho@gmail.com (N.K.); charalamposvoros@hotmail.com (C.V.); vryschri@hotmail.com (C.V.); stavros.athanasiou@gmail.com (S.A.);; 2 Unit of Obstetrics and Gynecology, “Paolo Giaccone” Hospital, Department of Health Promotion, Mother and Child Care, Internal Medicine and Medical Specialties (PROMISE), University of Palermo, 901 27 Palermo, Italy; mascellinog@gmail.com; 3 3rd Department of Obstretrics and Gyneocology, Attikon Hospital, National and Kapodistrian University of Athens, 124 62 Athens, Greece; dsioutis@gmail.com

**Keywords:** vNOTES, malignancies, gynecology

## Abstract

**Aim:** The purpose of this study was to investigate the safety, efficacy, and clinical outcomes of the vaginal natural orifice transluminal endoscopic surgery (vNOTES) technique in patients suffering from gynecological cancer. **Methods:** A systematic review of the literature was conducted from inception to October 2025 following the PRISMA guidelines. PubMed, Google Scholar, and the Cochrane Library were searched for studies investigating vNOTES in gynecological malignancies. Study quality was evaluated using the Newcastle–Ottawa Scale, the National Institute of Health and the Joanna Briggs Institute critical appraisal tools. **Results:** The search identified 11 observational cohort studies, 28 case series, and 22 case reports. A total of 926 patients with suspected or confirmed gynecologic malignancies underwent surgery via vNOTES approach. The combination of hysterectomy, bilateral salpingo-oophorectomy, and sentinel lymph node biopsy represented the most commonly performed surgical procedure. Endometrial cancer was the most frequent oncological indication. The included studies evaluated the perioperative outcomes, including operative time, estimated blood loss, lymph node assessment, conversion rates and complications. **Conclusions:** The vNOTES approach appeared to be feasible and at least non-inferior to standard surgical treatments for patients with early-stage gynecologic malignancies. However, the small sample sizes and heterogeneity among studies limit the strength of the evidence and preclude definitive conclusions.

## 1. Introduction

Gynecological malignancies constitute one of the most prevalent neoplasms worldwide, imposing a substantial burden on patients’ quality of life and healthcare systems. According to the Global Cancer Observatory (GLOBOCAN), these cancers accounted for 1,473,427 new cases and 680,372 deaths globally in 2022 [1]. While laparotomy remains the mainstay of surgical management for gynecological malignancies, their significant clinicopathological heterogeneity, coupled with patient variability, can render such interventions particularly complex [2,3,4]. Specifically, endometrial cancer, as the most prevalent gynecological malignancy, is closely associated with obesity, which poses substantial challenges for perioperative outcomes [5,6]. Furthermore, particularly in cases of ovarian cancer, surgical intervention is often performed in patients pretreated with neoadjuvant chemotherapy, a setting that induces an immunocompromised state and thereby increases susceptibility to surgical site infection [7,8]. In cervical cancer, preoperative assessment of pelvic and para-aortic lymph node status is pivotal in determining whether the management strategy will consist of surgical resection or primary radiotherapy [9]. Moreover, minimizing recovery time is crucial in these oncological patients to ensure the timely initiation of adjuvant therapy [10].

Gynecologic oncologists continually strive to adopt minimally invasive surgical alternatives in order to mitigate morbidity, curtail hospital stays, and expedite recovery. The vaginal natural orifice transluminal endoscopic surgery (vNOTES) represents a novel access route to the peritoneal cavity, utilizing the natural orifice of the vagina [11]. This technique offers a hybrid approach that integrates vaginal surgery with conventional laparoscopy, merging the benefits of a scarless vaginal route with the enhanced intraperitoneal visualization afforded by laparoscopic techniques [12]. In recent years, the vNOTES technique has been increasingly adopted for a broad spectrum of benign gynecological conditions. Specifically, procedures including salpingectomy, oophorectomy, myomectomy, and hysterectomy have demonstrated safety and efficacy profiles comparable to those of conventional laparoscopic approaches [13,14]. Furthermore, several studies have underscored multiple advantages of vNOTES over conventional laparoscopy, including reduced postoperative pain, shorter hospitalization, accelerated recovery, lower complication rates, and superior cosmetic outcomes attributed to the avoidance of abdominal incisions [15].

The demonstrated efficacy of the vNOTES approach in benign gynecological conditions, in conjunction with the specific needs of gynecologic oncology patients, has prompted the exploration of its potential application in this population. The aim of this systematic literature review is to objectively outline the currently available clinical data concerning the safety and efficacy, as well as the limitations, of the vNOTES approach in patients with gynecological malignancies.

## 2. Materials and Methods

### 2.1. Protocol Registration

The protocol of this review has been registered in PROSPERO, an international database for prospectively registered systematic reviews. The registration number for this review is CRD420251062011.

### 2.2. Search Strategy

The literature search covered the period from database inception to October 2025 and was conducted in the following electronic databases: PubMed, Google Scholar, and the Cochrane Library to ensure comprehensive coverage and inclusion of grey literature by two independent reviewers (AMK, EK). The search included Medical Subject Headings (MeSH) terms, along with a combination of the following keywords: vaginal natural orifice transluminal endoscopic surgery, endoscopy, malignancy, cancer, and gynecology. No filters were applied.

### 2.3. Eligibility Criteria

All studies concerning the application of the vNOTES technique in patients with suspected or confirmed gynecologic malignancies were evaluated based on their title, abstract, and full-text content. Furthermore, the references of all studies were evaluated for further citations.

All retrieved articles were independently assessed for eligibility by two reviewers (AMK, EK). The review considered retrospective and prospective observational studies, while excluding studies on non-gynecological procedures, review articles, publications in Hungarian and Chinese, ongoing studies and in vitro research. Additionally, studies investigating vNOTES for prophylactic purposes, including risk-reducing hysterectomy in asymptomatic patients with genetic predispositions to gynecological malignancies (e.g., Lynch syndrome, BRCA mutations), were not included.

Finally, this study was conducted following the Preferred Reporting Items for Systematic Reviews and Meta-Analysis (PRISMA) guidelines [16]. Supplementary Figure S1a,b present the comprehensive PRISMA 2020 Checklist.

### 2.4. Quality Assessment of the Included Studies

Quality assessment of the included observational cohort studies was performed using the Newcastle–Ottawa Scale (NOS). The NOS evaluates studies according to three key domains: Selection, Comparability, and Outcome. Each study could be awarded a maximum of nine stars. Studies achieving a score of 7–9 were classified as high quality, those scoring between 4 and 6 as moderate quality, and those with a score of 0–3 as low quality, indicating a high risk of bias [17].

The National Institute of Health (ΝΙΗ) Quality Assessment Tool for Case Series Studies was used to evaluate the methodological quality of the observational case series. This instrument assesses nine methodological criteria, with each component rated as “Yes,” “No,” “Not reported,” or “Not applicable.” The final assessment is qualitative, based on the reviewer’s subjective judgment, and classifies studies into three categories: good, fair, and poor, according to the distribution of “Yes” and “No” responses to critical criteria, representing the corresponding degree of risk of bias [18].

In addition, the methodological rigor of the case report studies was assessed using the Joanna Briggs Institute (JBI) Critical Appraisal Checklist for Case Reports. This checklist consists of eight questions evaluating the methodological quality of each study, with responses recorded as “Yes,” “No,” “Unclear,” or “Not applicable,” providing a qualitative assessment that categorizes studies as high, moderate, or low quality according to the number of criteria fulfilled [19].

Disagreements regarding quality assessments were resolved by involving a third reviewer (DZ).

### 2.5. Data Extraction

Details were independently collected by two authors (AMK, EK), including variables as the year of publication, hospital setting, study methodology, sample size, type of intervention, patient demographics, type of cancer, and clinical outcomes The results were organized and presented based on study design to improve clarity and consistency in data presentation.

However, no additional statistical synthesis was performed in the present review due to methodological heterogeneity. All variables are presented descriptively, with summary measures reported only in terms of the range of mean and/or median values, as available in the original studies.

## 3. Results

**Figure 1:**
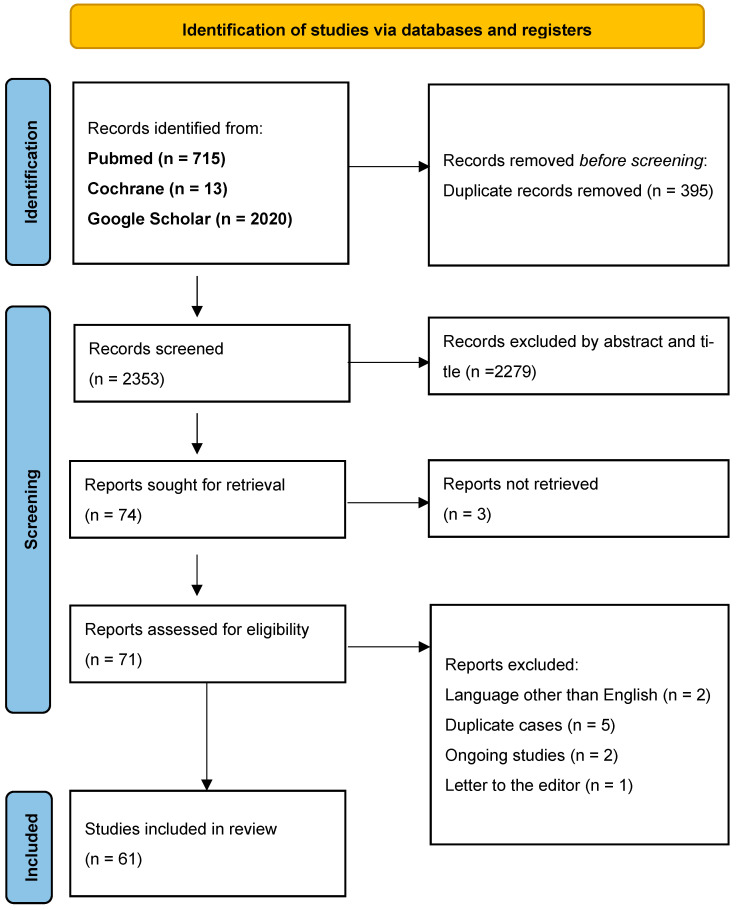
PRISMA 2020 flow diagram of identification, screening, and inclusion of relevant studies.

According to the literature, the use of the vNOTES technique in the management of gynecologic cancer has been documented in eleven observational cohort studies [20,21,22,23,24,25,26,27,28,29,30], 28 case series [31,32,33,34,35,36,37,38,39,40,41,42,43,44,45,46,47,48,49,50,51,52,53,54,55,56,57,58], and 22 case reports [59,60,61,62,63,64,65,66,67,68,69,70,71,72,73,74,75,76,77,78,79,80], with the earliest publication dating back to 2008. Figure 1 presents the PRISMA flow diagram, outlining the process of article identification, screening, and selection for inclusion in this analysis.

Table 1 summarizes the demographic characteristics of all included studies.

### 3.1. Observational Cohort Studies

A total of eleven comparative cohort studies (ten retrospective and one prospective) comprising 906 patients who underwent gynecological procedures were included. The extracted perioperative and surgical outcomes are summarized in Table 2. Among these, 601 (66.33%) patients were treated via vNOTES approach, including 311 (34.32%) cases of gynecological malignancies. Notably, one study involved a cohort of 100 patients, evaluating vNOTES hysterectomy (vNOTES-H) and vaginal hysterectomy (VH) (*n* = 50 vNOTES-H vs. *n* = 50 VH); however, the specific number of malignancy cases in this group was not explicitly reported [29].

The included cohort studies were conducted in tertiary care centers across Turkey, Singapore, China, and France between 2021 and 2025. The overall patient demographics varied, with mean ages ranging from 37 years [23] to 62.5 years [21]. Among studies restricted to oncological patients, the vNOTES cohorts reported a median body mass index (BMI) ranging between 21 kg/m^2^ [23] and 31 kg/m^2^ [22].

Among the oncological cases treated via vNOTES, 285 (91.63%) patients had endometrial cancer or endometrial intraepithelial neoplasia [20,21,22,24,25,26,27,28,29,30], 19 (6.1%) had histologically confirmed ovarian malignancies [23], and 7 (2.25%) patients were diagnosed with early-stage cervical cancer [28]. The most commonly performed vNOTES oncological procedure was total hysterectomy, frequently combined with bilateral, and less commonly unilateral, salpingo-oophorectomy. Lymph node assessment included sentinel lymph node dissection (SLND) in 211 (67.84%) cases of early-stage endometrial cancer [20,21,25,26,30], while pelvic lymph node dissection (PLND) was performed in three (0.96%) cases of ovarian cancer [23]. Furthermore, one (0.32%) case of endometrial cancer involved both PLND and para-aortic lymph node sampling [28]. Finally, eight (2.57%) patients with ovarian cancer underwent omentectomy, including two (0.64%) who underwent interval debulking surgery via the vNOTES approach [23]. A total of 208 (22.95%) oncological patients across studies had endometrial cancer and underwent alternative minimally invasive surgical (MIS) techniques, including single-port laparoscopy (SPLS), conventional laparoscopy (CL), multiport laparoscopy (ML), robotic surgery or VH. In the study by Merlier et al., patients with grade 1 endometrioid adenocarcinoma were included among the study population; however, the exact number of patients with benign versus malignant pathology was not specified [29].

Among the included studies, estimated blood loss (EBL) was reported in six studies, none of which detected a statistically significant difference between patients undergoing vNOTES and those treated with other MIS approaches [22,24,25,26,29,30]. A statistically significant reduction in operative time was reported in only one study, with an advantage for the vNOTES approach over SPLS [20]. In addition, five studies demonstrated that vNOTES was associated with statistically significant lower postoperative pain scores at 6, 12, and 24 h compared with other MIS approaches [20,22,24,25,26], whereas one study reported no significant difference between vNOTES and CL [30]. With respect to the length of hospital stay, four studies reported comparable outcomes between vNOTES and other MIS techniques [20,22,24,29], while three studies showed a statistically significant reduction favoring the vNOTES approach [25,26,30]. Sentinel lymph node (SLN) detection rates were evaluated in three studies and were not found to differ significantly between vNOTES and other surgical approaches [25,26,30]. Regarding the latter, Arkan et al. evaluated indocyanine green (ICG) versus methylene blue for SLN mapping via vNOTES in endometrial cancer staging, showing that ICG significantly increased both the detection rate and the number of SLNs identified per patient [21]. In terms of safety, no significant differences were observed in intra- and postoperative complications [22,24,25,26,29,30] or conversion rates [22,24,26,29,30]. Only a single case of intraoperative hemorrhage in vNOTES group, with an EBL of approximately 1000 mL, was reported by Fong et al. [23]. Conversion to CL was required in two cases of vNOTES, one due to a large uterus [26] and another due to failed bilateral pelvic sentinel lymph node mapping [30]. Apart from the aforementioned case of significant blood loss and the two conversions to CL, no major intraoperative or postoperative complications were reported in any of the studies.

Table 3 provides an overview of the risk-of-bias assessment for the included studies; all studies were considered moderate quality and risk of bias (NOS 4–6).

**Table 1:** Demographic Characteristics of the Included Studies Evaluating Vaginal Natural Orifice Transluminal Endoscopic Surgery (vNOTES) for Gynecologic Procedures in Patients with Malignancy.

Author, Year, Reference	Study Design	Country, Duration of Study	Inclusion Criteria	Exclusion Criteria
Gungorduk K. et al., 2025 [20]	Observational cohort study	Turkey, January 2020 to June 2024	Hysterectomy + BS ± SO/SLND	Deep endometriosis Pelvic masses suggestive ovarian cancer
Arkan K. et al., 2025 [21]	Observational cohort study	Turkey, NR	EC: FIGO st I-II	Suspected metastatic disease Prior pelvic or para-aortic LND Known allergies to the ICG or MB Cases that required conversion to CL (*n* = 12)
Şimşek E. et al., 2025 [22]	Observational cohort study	Turkey, January 2023 to June 2024.	Patients > 18 y EC Free of any other malignant pelvic disease Without previous pelvic/abdominal RT Uteruses that were large enough to be removed vaginally in both surgical methods	Previous RS for para-aortic LND Deep endometriosis
Fong K.Y. et al., 2025 [23]	Observational cohort study	Singapore, May 2021 to September 2024	Histology-proven OC	N/A
Mat E. et al., 2024 [24]	Observational cohort study	Turkey, January 2019 to November 2020	Low grade EC or EIN ^a^	Contraindications for pneumoperitoneum, dorsal lithotomy position, general anesthesia Severe cardio pulmonary renal disease History of colorectal surgery Blood clotting disorders History of pelvic RT Tubo-ovarian abscesses. Douglas pouch obliteration.
Comba C. et al., 2024 [25]	Observational cohort study	Turkey, February 2021 to June 2023	Age: 30–85 y Diagnosis of EC No distant organ metastases No medical contraindications to surgery SLND	Systemic lymphadenectomy for EC Presence of distant organ metastases
Deng L. et al., 2023 [26]	Observational cohort study	China, January 2021 to May 2022	≥18 y EC St I in MRI No tumor mass > 2 cm Patient accepted MIS Biochemical exams normal ECOG ≤ 1 No other malignant tumor in the last 5 y	Previous pelvic or abdominal RT Uterus > 12 cm Contraindications to surgery Inadequate FU
Bouchez M.C. et al., 2023 [27]	Observational cohort study	France, February 2020 to January 2022	All patients requiring a hysterectomy	Endometriosis Cancer ^b^
Mei Y. et al., 2023 [28]	Observational cohort study	China, February 2020 to January 2022	NR	Obliteration cul de sac Deep endometriosis Late st CaCx/EC History of multiple prior open abdominal operations
Merlier M. et al., 2022 [29]	Observational cohort study	France, March 2019 to November 2020	All patients requiring hysterectomy	Endometriosis Oncological indications c
Wang Y. et al., 2021 [30]	Observational cohort study	China, August 2017 to May 2020	Low-grade (1 or 2) EC Lesion confined to the uterine body Tumor <4 cm in diameter Lesion not involving the cervix No intraperitoneal metastasis Surgical staging with SLND, including vNOTES and LAP	N/A
Nef J. et al., 2025 [31]	Case series study	Switzerland, May 2020 to November 2024	≥65 y	N/A
Gungorduk K. et al., 2025 [32]	Case series study	Turkey (multi-center), January 2023 to May 2025	vNOTES hysterectomy ± SO	UP > st I POPQS History of endometriosis or PID Severe renal failure Severe cardiopulmonary disease Previous colorectal surgery Contraindications to general anesthesia or Trendelenburg position
Hanedan C. et al., 2025 [33]	Case series study	Turkey, NR	vNOTES hysterectomy Uterine weight ≥ 280 g	Uterine weight < 280 g No consent
Kellerhals G. et al., 2025 [34]	Case series study	Switzerland, October 2021 to August 2024	NR	N/A
Yang Q. et al., 2025 [35]	Case series study	Huston, USA, June 2019 to August 2024	RA-vNOTES using the Da Vinci Xi robotic system	N/A
Tan R.C.A. et al., 2025 [36]	Case series study	Singapore, April 2021 to May 2024	vNOTES hysterectomy	N/A
Simsek E. et al., 2024 [37]	Case series study	Turkey, January 2022 to June 2024	EC confined to the uterus	Prior gynecological/abdominal malignancy surgery Deep endometriosis Big uterus for vaginally removal
Baekelandt J. et al., 2024 [38]	Case series study	Switzerland, USA, Brazil and Belgium, March 2016 to May 2023	EC	N/A
Matak L. et al., 2024 [39]	Case series study	NA, August 2023 to October 2023	NR	N/A
Huber D. et al., 2024 [40]	Case series study	Switzerland, October 2021 to November 2023	EC/CAH confined to the uterus No metastases Indication to surgical staging with SLND SLND by retroperitoneal vNOTES	N/A
Zarragoitia J. et al., 2024 [41]	Case series study	Spain, 2022 to 2023	NR	N/A
Burnett A.F. et al., 2024 [42]	Case series study	Arkansas, Mississippi, Belgium, 2017 to 2023	BMI ≥ 40 kg/m2 for gynecologic surgery and consent for vNOTES	Obliteration of the cul de sac due to prior low colorectal surgery Prior PID Deep endometriosis
Hurni Y. et al., 2023 [43]	Case series study	Switzerland, May 2020 to April 2023	BMI ≥ 30 kg/m2 and VNOTES for gynecological indication	Concomitant PSLNB, infracolic omentectomy or appendectomy
Hurni Y. et al., 2023 [44]	Case series study	Switzerland, May 2020 to April 2023	Early stage of disease	Suspicion of advanced stage disease History of perineal/rectal surgery History of pelvic RT Deep endometriosis Active PID
Mat E. et al., 2023 [45]	Case series study	Turkey, June 2021 to December 2021	NR	Any contraindication for pneumoperitoneum or dorsal lithotomy position or general anesthesia Sepsis Severe renal failure Severe cardiopulmonary disease History of colorectal surgery Suspicion of uterine sarcoma Blood coagulation disorders History of pelvic RT Tubo-ovarian abscesses Obliteration of the cul de sac
Burnett A.F. et al., 2023 [46]	Case series study	NR, NR	SLND in gynecologic malignancies	N/A
Kale A. et al., 2022 [47]	Case series study	Turkey, January 2019 to April 2021	BMI > 30 kg/m^2^	Any contraindication for pneumoperitoneum or dorsal lithotomy position or general anesthesia Virginity or a narrow vagina Suspicion of deep endometriosis Obliteration of the cul-de-sac Previous rectovaginal surgery Large uterus Sepsis Serious renal failure Severe cardiopulmonary disorder Blood coagulation disorders
Huang L. et al., 2022 [48]	Case series study	China, April 2018 to May 2021	NR	Intolerability of the procedure Acute infection stage Deep venous thrombosis Hypercoagulability Liver or kidney dysfunction Mental illness History of rectal surgery Suspected deep endometriosis or severe adhesions Virginity Pregnancy
Lee C.-L. et al., 2022 [49]	Case series study	Taiwan, January 2014 to December 2020	Age of 20–80 y EC, st I, grade 1–2, endometroid histopathology type MRI without positive lymph nodes	Virginity or narrow vagina History of multiple abdominopelvic surgeries BMI > 42 kg/m2 History of any previous incomplete surgery History of deep endometriosis surgery Suspicion of the cul de sac obliteration No FU and had incomplete adjuvant therapy
Huber D. et al., 2022 [50]	Case series study	Switzerland, October 2021 to February 2022	EC, grade 1 or 2 or CAH No evidence of metastases Indication to surgical staging with SLND SLND by retroperitoneal vNOTES	N/A
Comba C. et al., 2022 [51]	Case series study	Turkey, NR	NR	N/A
Mat E. et al., 2021 [52]	Case series study	Turkey, November 2018 to May 2019	NR	Any contraindication for pneumoperitoneum or dorsal lithotomy position or general anesthesia Sepsis Serious renal failure Severe cardiopulmonary disorder Menstrual period/pregnancy Blood coagulation disorders Obliteration of the cul de sac
Mat E. et al., 2021 [53]	Case series study	Turkey, January 2019 to June 2019	Extreme obese patients with early-stage EC	Any contraindication for pneumoperitoneum or dorsal lithotomy position or general anesthesia Sepsis Serious renal failure Severe cardiopulmonary disorder Blood coagulation disorders Obliteration of the cul de sac Previous rectovaginal surgery Any laparotomy or laparoscopy involving the sigmoid or the rectum Large uterus requiring morcellation Contraindication to Trendelenburg position
Lowenstein L. et al., 2020 [54]	Case series study	Israel, Belgium, November 2018 to August 2019	NR	N/A
Karkia R. et al., 2019 [55]	Case series study	UK, January 2018 to December 2018	Benign uterine pathology or EC, st I, grade 1 For EC, hysterectomy eligibility was verified by MRI and multidisciplinary team discussion	History of surgery to the rectovaginal pouch Deep endometriosis ≥2 cesareans Patients with UP were excluded, as conventional VH was preferred
Tantitamit T., 2019 [56]	Case series study	Taiwan, NR	EC, st I	N/A
Kaya C. et al., 2018 [57]	Case series study	Turkey, January 2017 to May 2017	No contraindication for pneumoperitoneum or the Trendelenburg position No fixed uterus or nodularity in the cul de sac on bimanual PE No history of PID, pelvic abscess or endometriosis	N/A
Lee C.-L. et al., 2014 [58]	Case series study	Taiwan, NR	Eligible for laparoscopic staging	N/A
Zhang C. et al., 2025 [59]	Case report	China, NR	NR	N/A
Baekelandt J. et al., 2024 [60]	Case report	Belgium, NR	NR	N/A
Can B. et al., 2024 [61]	Case report	Turkey, NR	NR	N/A
Ng W. et al., 2024 [62]	Case report	Singapore, NR	NR	N/A
Erkilinc S. et al., 2024 [63]	Case report	Turkey, NR	NR	N/A
Guevara R. et al., 2024 [64]	Case report	Spain, NR	NR	N/A
Couso A. et al., 2024 [65]	Case report	Spain, NR	NR	N/A
Baekelandt J. et al., 2023 [66]	Case report	NR	NR	N/A
Kita M., et al., 2023 [67]	Case report	Japan, NR	NR	N/A
Li Y. et al., 2022 [68]	Case report	China, NR	NR	N/A
Hurni Y. et al., 2022 [69]	Case report	Switzerland, NR	NR	N/A
Mathey M.-P. et al., 2022 [70]	Case report	Switzerland, January 2021	NR	N/A
Hurni Y. et al., 2022 [71]	Case report	Switzerland, October 2021	NR	N/A
Lim Y.H. et al., 2022 [72]	Case report	Singapore	NR	N/A
Comba C. et al., 2021 [73]	Case report	Turkey, NR	NR	N/A
Kita M. et al. 2021 [74]	Case report	Japan, NR	NR	N/A
Ju Y.Y. et al., 2021 [75]	Case report	Korea, NR	NR	N/A
Badiglian-Filho L., 2020 [76]	Case report	Brazil, NR	NR	N/A
Oh S.H., 2019 [77]	Case report	Republic of Korea, NR	NR	N/A
Htay W.T., 2019 [78]	Case report	Taiwan, NR	NR	N/A
Leblanc E., 2016 [79]	Case report	France, NR	NR	N/A
Zorrón R. et al., 2008 [80]	Case report	Brazil, NR	Low ASA risk Eligible to transvaginal approach instead of LAP	N/A

Abbreviations: BS, bilateral salpingectomy; SO, salpingo-oophorectomy; SLND, sentinel lymph node dissection; NR, not reported; ICG, indocyanine green; MB, methylene blue; EC, endometrial cancer; FIGO, International Federation of Gynecology and Obstetrics; st, stage; LND, lymph node dissection; CL, conventional laparoscopy; y, years; RT, radiotherapy; RS, robotic surgery; OC, ovarian cancer; N/A, not applicable; EIN, endometrial intraepithelial neoplasia; ^a^ From preoperative endometrial sampling, with lesion confined to uterus. MRI, magnetic resonance imaging; cm, centimeters; MIS, minimally invasive surgery; ECOG, Eastern Cooperative Oncology Group Performance Status; FU, follow-up; *^b^* Except grade 1 endometrioid adenocarcinoma. CaCx, cervical cancer; ^c^ Except grade 1 endometrioid adenocarcinoma. vNOTES, vaginal natural orifice transluminal endoscopic surgery; LAP, laparoscopy; UP, uterine prolapse; POPQS, pelvic organ prolapse quantification system; PID, pelvic inflammatory disease; g, grams; RA-vNOTES, Robot-Assisted Vaginal Natural Orifice Transluminal Endoscopic Surgery; CAH, complex atypical hyperplasia; BMI, body mass index; kg, kilogram; m, meter; PSLNB, pelvic sentinel lymph node biopsy; VH, vaginal hysterectomy; PE, pelvic exam.

**Table 2:** Perioperative and Surgical Outcomes of the Included Studies Evaluating Vaginal Natural Orifice Transluminal Endoscopic Surgery (vNOTES) for Gynecologic Procedures in Patients with Malignancy.

Author, Year, Reference	Sample (Cases/Controls)-Diagnosis	vNOTES Intervention (Cases/Controls)	Patients Characteristics (Cases/Controls)	Outcomes
Gungorduk K. et al., 2025 [20]	Total: *n* = 121 vNOTES (*n* = 58) vs. SPLS (*n* = 63) Oncological indications: *n* = 30 EC (*n* = 30) vNOTES (*n* = 14) vs. SPLS (*n* = 16)	Hysterectomy + USO/BSO ± SLND ^a^	Age, mean ± SD: 54.9 ± 6.3 vs. 55 ± 8.2 y BMI, mean ± SD: 30.6 ± 3.4 vs. 30.8 ± 5.5 kg/m^2^	vNOTES group: Shorter operative time Lower pain scores at 6 h, 12 h and 24 h No significant difference in: Hospital stay SFI 3 months Dyspareunia
Arkan K. et al., 2025 [21]	Total: *n* = 80 ICG (*n* = 40) vs. MB (*n* = 40) EC, st I-II: *n* = 80	Hysterectomy + BSO + SLND using ICG/MB	Age, mean ± SD: 62.5 ± 8.1 vs. 61.8 ± 7.5 y BMI, mean ± SD: 28.7 ± 4.5 vs. 29.1 ± 4.2 kg/m^2^	ICG group: Higher SLN detection rate Higher mean SLNs per patient No significant difference in: Βilateral SLN detection rate Operative time EBL Transfusion requirements Complication rates
Şimşek E. et al., 2025 [22]	Total: *n* = 76 vNOTES (*n* = 24) vs. RS (*n*= 52) EC: *n* = 76	vNOTES: hysterectomy + BSO. Robotic surgery is NR.	Age, mean ± SD: 56.5 ± 3.5 vs. 62 ± 11 y BMI, mean ± SD: 31 ± 2.25 vs. 31 ± 4.5 kg/m^2^	vNOTES group: Lower pain scores at 12 h No significant difference in: Operative time EBL Hospital stay No conversion to CL/Laparotomy Intraoperative complications Postoperative complications
Fong K.Y. et al., 2025 [23]	Total: *n* = 19 OC: *n* = 19	Procedures ^b^: primary staging surgery (*n* = 12), fertility-sparing surgery (*n* = 4), restaging surgery (*n* = 1) and IDS (*n* = 2)	Age, median (range): Primary staging surgery group: 62 (54–67.5) y Fertility-sparing surgery group: 37 (33–38) y BMI, median (range): Primary staging surgery group: 25.9 (22.7–30.0) kg/m^2^ Fertility-sparing surgery group: 21 (20.6–24.2) kg/m^2^	Primary staging group: EBL: 100 (100–200) mL Operative time: 135 [114, 221] min 1 intraoperative complication [high EBL (1000 mL)] Hospital stay: 2.5 (2–3) days FU time: 27.6 (7.8–31.4) months Fertility-sparing group: EBL: 75 (50–125) mL Operative time: 105 (91–108) min No intraoperative complications Hospital stay: 2 (2) days FU time: 11.7 (7.1–17.7) months
Mat E. et al., 2024 [24]	Total: *n* = 45 vNOTES (*n* = 29) vs. CL (*n* = 16) Low grade EC/EIN: *n* = 45	Hysterectomy + BSO	Age, mean ± SD: 59 ± 9.2 vs. 58 ± 12.8 y BMI, median (range): 29.2 (25.3–41.6) vs. 28.2 (23.5–31.1) kg/m^2^	vNOTES group: Lower pain scores at 6, 12 and 24 h. Lower need for analgesics No significant difference in: Operative time The decrease in Hb levels Hospital stay Either vNOTES or CL group: Conversions Damage to adjacent organs Hematoma Re-operations No metastasis or recurrence in 3 y
Comba C. et al., 2024 [25]	Total: *n* = 57 vNOTES (*n* = 19) vs. CL (*n* − 38) EC: *n* = 57	Hysterectomy + BSO + SLND	Age, mean ± SD: 59.4 ± 11.6 vs. 59.8 ± 9.9 y BMI, median (range): 29 (24–38) vs. 32 (19–54) kg/m^2^	vNOTES group: Lower pain scores Shorter hospital stay No significant difference in: Operative time EBL Number of SLN SLN detection rate: vNOTES 94.8% and CL 100%. Complications The decrease in Hb levels ICU Either vNOTES or CL group: No recurrence
Deng L. et al., 2023 [26]	Total: *n* = 120 vNOTES (*n* = 57) vs. ML (*n* = 63) EC: *n* = 120	Hysterectomy + BSO + SLND	Age, median ± SD: 51.46 ± 7.83 vs. 52.52 ± 8.47 y BMI, median ± SD: 26.25 ± 3.09 vs. 25.76 ± 4.10 kg/m^2^	vNOTES vs. ML: PSDR: 94.73% vs. 96.82% The bilateral detection rate: 82.46 vs. 84.13% The side specific mapping: 88.6 vs. 90.48% Same number of SLN vNOTES: Lower pain scores Shorter hospital stay 1 ML convertion (large uterus) No significant difference in: Operative time EBL Either vNOTES or ΜL group: No complications Same medical costs
Bouchez M.C. et al., 2023 [27]	Total: *n* = 200 Oncological indications: *n* = 10 EC, grade 1 (*n* = 10) BMI < 30 (*n* = 6) vs. BMI ≥ 30 (*n* = 4)	Hysterectomy ± salpingectomy or BSO	Age, mean ± SD: 47.30 ± 7.59 y (total) 47.3 ± 7.9 y (BMI < 30) vs. 47.1 ± 6.5 y (BMI ≥ 30) BMI, median (range): 26.2 (15.90–48.40) (total) 24.3 (21.82–27.77) (BMI < 30) vs. 34.0 (31.68–37.08) (BMI ≥ 30) kg/m^2^	Total study population: BMI ≥ 30 group: Longer operative Longer hospital stay No significant difference in: Complications Intraoperative conversion EBL
Mei Y. et al., 2023 [28]	Total: *n* = 14 GR-vNOTES (*n* = 5) vs. TR-vNOTES (*n* = 9) Oncological indications: *n* = 8 GR-vNOTES (*n* = 2) ^c^ vs. TR-vNOTES (*n* = 6) ^d^	Hysterectomy + salpingectomy ± bilateral oophorectomy ± pelvic lymphadenectomy ± para-aortic lymph node sampling ^f^	Total study population: Age, mean ± SD: GR-vNOTES 44.6 ± 6.5 vs. TR-vNOTES 48.5 ± 6.9 y BMI, median ± SD: GR-vNOTES 21.4 ± 3.3 vs. TR-vNOTES 22.4 ± 1.6 kg/m^2^	Total study population: No significant difference in: Operative time EBL Hospital stay Pain scores on the operative day, 1 day and 2 day No conversion to CL Complications Patients for oncology indication: No recurrence or metastasis in 6 months
Merlier M. et al., 2022 [29]	Total: *n* = 100 vNOTES (*n* = 50) vs. VH (*n* = 50) Oncological indications: *n* = unidentified EC, grade 1 (*n* = unidentified)	Hysterectomy ± salpingectomies ± adnexectomies ^g^	Total study population: Age, mean ± SD: vNOTES 48.6 ± 7.4 vs. VH 49.5 ± 8.5 y BMI, median ± SD: vNOTES 27.5 ± 6.4 vs. VH 25.6 ± 5.2 kg/m^2^	Total study population: No significant difference in: Hospital stay The rate of outpatient success rate EBL Complications
Wang Y. et al., 2021 [30]	Total: *n* = 74 vNOTES (*n* = 51) vs. CL (*n* = 23) EC, low grade: *n* = 74	Hysterectomy + SLND	Age, median (range): 53 (48–55) vs. 52 (49–56) y BMI, median (range): 24.3 (22.7–26.7) vs. 24.4 (22.1–26.7) kg/m^2^	No significant difference in: Operative time EBL Post operative complications Postoperative pain scores at 12 and 24 h SLND outcomes Intraoperative complications: 0 Conversion to CL:1 h vNOTES group: Lower hospital stay
Nef J. et al., 2025 [31]	Total: *n* = 119 Oncological indications: *n* = 44 EC (*n* = 39), CaCx (*n* = 2), carcinosarcoma (*n* = 1), LGSOC(*n* = 1), BOT (*n* = 1)	Hysterectomy + USO/BSO ± bilateral PSLND ± infracolic omentectomy	Total study population: Age, mean ± SD: 72.5 ± 5.3 y BMI median ± SD: 27.4 ± 6.4 kg/m^2^	Total amount of the study: Operative time: 81.6 (15–221) min EBL: 66.5 (0–500) mL Conversion to laparoscopy: 3.4% No conversion to laparotomy Intraoperative complications: 11.8% Postoperative complications: 16,8% Postoperative opioid: 13.5% Hospital stay: 2.8 (1–8) days
Gungorduk K. et al., 2025 [32]	Total: *n* = 685 Oncological indications: *n* = 130 EC/endometrial hyperplasia (*n* = 130)	Hysterectomy + US/BS/USO/BSO + SLND	Total study population: Age, mean ± SD: 50.7 ± 9.3 y BMI, mean ± SD: 29.0 ± 5.5 kg/m^2^	Total amount of the study: Operative time: 72.4 ± 40.2 min The decrease in Hb levels: 1.4 ± 0.1 g/dL The mean uterine weight: 204 ± 145 g Intraoperative complications: *n* = 12 Postoperative complications: *n* = 10 Conversions to laparoscopy: *n* = 6 Pain scores at 24 h: 2.7 ± 0.8 Hospital stay: 2.3 ± 1.4 days
Hanedan C. et al., 2025 [33]	Total: *n* = 46 Oncological indications: *n* = 3 EC (*n* = 3)	Hysterectomy + BS ± oophorectomy ± SLND	Total study population: Age, median (range): 54 (40–74) y BMI, median (range): 31 (21–51) kg/m^2^	Total study population: Operative time: 56 (35–95) min Uterine weight: 410 (280–1036) g Preoperative Hb: 13.1 (8.6–15.9) g/dL Postoperative Hb: 11.7 (8.8–14.4) g/dL Hospital stay: 30 (16–72) h Conversion to laparoscopy: *n* = 2 Intraoperative complication: *n* = 1 Postoperative complication: *n* = 1
Kellerhals G. et al., 2025 [34]	Total: *n* = 11 OC, early stage: *n* = 7 BOT: *n* = 4	USO/BSO + peritoneal washing ± infracolic omentectomy ± pelvic peritonectomy ± rectal mesenteric implant excision ± total hysterectomy	Age, median (range): 47 (27–81) y BMI, median (range): 28.1 (22,4–39.2) kg/m^2^	Operative time: 70 (35–138) min EBL: 50 (10–100) mL No conversions No intraoperative complications ^i^ Postoperative complications: 27.3% ^j^
Yang Q. et al., 2025 [35]	Total: *n* = 298 Oncological indications: *n* = 2 EC (*n* = 2)	Hysterectomy ± USO/BSO	Total study population: Age, median (range): 41 (36–46) y BMI, median (range): 29 (24–35) kg/m^2^	Total study population: Operative time: 138 (116–167) min EBL: 50 (25–50) mL Discharge within 24 h: 69.13% Conversion: 1.01% Complication rate: 16.78%
Tan R.C.A. et al., 2025 [36]	Total: *n* = 176 Oncological indications: *n* = 3 CIN/low-grade CaCx and adenomyosis (*n* = 3)	Hysterectomy ± salpingo-oophorectomy/salpingectomy	Total study population: Age, mean ± SD: 52.3 ± 9.8 y BMI, mean ± SD: 25.6 ± 5.2 kg/m^2^	Total study population: Operative time: 109.7 ± 44.3 min Uterine weight: 243.6 ± 198.7 g Hospital stay: 1.6 ±1.0 days EBL: 163.8 ± 185.5 mL Intraoperative complication: *n* = 1 Postoperative complications: *n* = 5
Simsek E. et al., 2024 [37]	Total: *n* = 24 patients EC: *n* = 24	Hysterectomy + BSO + retroperitoneal SLND with ICG.	Age, mean (range): 56.5 (51–65) y BMI, median (range): 31 (29–38) kg/m^2^	Operative time: 125 min EBL: 70 mL Pain scores at 12 h: 2 Hospital stay: 1 day Detection of SLN: 23/24 No conversions No intraoperative complications
Baekelandt J. et al., 2024 [38]	Total: *n* = 64 EC: *n* = 64	Hysterectomy + BSO + retroperitoneal SLN	Age, mean (range): 69.5 (45–89) y BMI, median (range): 26 (16–48) kg/m^2^	Operative time: 126 min. EBL: 80 mL Bilateral SLN: 97% and unilaterally: 3% k Hospital stay: 2 days Conversion to laparoscopy: *n* = 1 Reintervention: *n* = 1 Pain scores: 1
Matak L. et al., 2024 [39]	Total: 4 EC, grade 1:4	Hysterectomy + adnexectomy + SLN mapping with ICG	Age, mean (range): 67 (53–82) y BMI, mean: 28.45 kg/m^2^	Operative time: 169 (150–200) min Hemoglobin value decrease: 14% No complications Hospital stay: 2 days The mean number of lymph nodes: 12.5
Huber D. et al., 2024 [40]	Total: *n* = 34 Oncological indications: *n* = 32 EC (*n* = 32)	vNOTES unilateral/bilateral SLND + vNOTES or conventional VH + BSO.	Total study population: Age, mean (range): 68 (45–87) y BMI, median (range): 27.3 (16–48.9) kg/m^2^	Total study population: 97.1% successful procedure SLND Conversion to CL: *n* = 1 The bilateral SNL detection rate 91.2, and the unilateral detection rate SNL: 5.9%.
Zarragoitia J. et al., 2024 [41]	Total: *n* = 54 EC/CaCx, early stage and premalignancy: *n* = 54	vNOTES procedure ± bilateral SLND (*n* = 26)	NR	Operative time: 130 min EBL: 70 mL No intraoperative complications No blood transfusions Hospital stay: 1 day Complication: *n* = 1
Burnett A.F. et al., 2024 [42]	Total: 103 Oncological indications: *n* = 46 EC/endometrial hyperplasia (*n* = 46)	Hysterectomy ± adnexae ± BSO ± pelvic nodes	Total study population: Group BMI 40–49.9: Age, mean (range): 49.6 (26–73) y BMI, mean (range): 45.7 (40–49.6) kg/m^2^ Group BMI 40–49.9: Age, mean (range): 55.7 (35–72) y BMI, mean (range): 54.3 (50–62) kg/m^2^	Total study population: Group of BMI 40–49.9: Conversion to laparoscopy: *n* = 2 Conversion to laparotomy: *n* = 4 Operation time: 82 (30–232) min EBL: 80 (10–400) mL Outpatient: *n* = 47 Observation: *n* = 32 Two days hospital stay: *n* = 2 3–4 days hospital stay: *n* = 3 ^l^ Group of BMI 40–49.9: Conversion to laparoscopy: *n* = 0 Conversion to laparotomy: *n* = 1 Operative time: 114 (50–223) min EBL: 104 (65–402) mL Outpatient: *n* = Observation: *n* = 11 Two days hospital stay: *n* = 2 3–4 days hospital stay: *n* = 0 ^l^
Hurni Y. et al., 2023 [43]	Total: *n* = 79 Oncological indications: *n* = 3 EC (*n* = 3) ^m^	Hysterectomy ± US/BS ± USO/BSO ± BPSLND ± infracolic omentectomy ± appendectomy	Total study population: Age, median (range): 51 (32–79) y BMI, median (range): 35.2 (30.1–49.4) kg/m^2^	Total study population: Operative time: 91 (44–193) min Conversion to CL: *n* = 4 No conversion to laparotomy Intraoperative complications: *n* = 3 Postoperative complications: *n* = 6 Hospital stay: 2 (1–7) days Pain scores at 12 h, 24 h and 48 h were 1, 2 and 1 respectively
Hurni Y. et al., 2023 [44]	Total: *n* = 18 EC, high risk: *n* = 4 SAM: *n* = 14	Hysterectomy + USO/BSO + infracolic omentectomy ± peritoneal biopsies ± appendectomy ± BPSLND	Age, median (range): 59 (30–81) y BMI, median (range): 23.8 (16–39.2) kg/m^2^	Operative time: 80 (35–178) min EBL: 50 (10–150) mL No conversion occurred ^n^ Hospital stay: 3 cases: one day and 15 cases: 2 (1–5) days Pain scores at 12 h, 24 h and 48 h: 0.5, 3 and 1 respectively No opioids needed No significant intraoperative complications Postoperative complication: *n* = 1 No reintervention
Mat E. et al., 2023 [45]	Total: *n* = 11 Oncological indications: *n* = 7 EC, grade 1 (*n* = 7)	Hysterectomy + BSO	Total study population: Age, mean ± SD: 75.91 ± 6.47 y ΒΜΙ, mean ± SD: 42.49 ± 8.77 kg/m^2^	Total study population: Operation time: 66.18 ± 25.69 min EBL: 43.64 ± 14.50 mL Hospital stay: 2.55 ± 1.21 days Pain scores at 6 h, 12 h and 24 h: 2.9, 2.0, 0.8 respectively
Burnett A.F. et al., 2023 [46]	Total: *n* = 58 Endometrial hyperplasia or uterine cancer: *n* = 54 CaCx: *n* = 4	Hysterectomy + BSO + BSLND	Age, mean (range): 67.3 (35–89) y BMI, mean (range): 27.2 (16–48) kg/m^2^	Operative time: 126 (64–270) min EBL: 98 (20–400) mL Complications *n* = 2 ^o^
Kale A. et al., 2022 [47]	Total: *n* = 81 Oncological indications: *n* = 26 EC, early stage (*n* = 22), peritoneal carcinomatosis (*n* = 3), gastric carcinoma (*n* = 1)	EC (*n* = 22): hysterectomy + BSO Ascites of unknown origin (*n* = 4): right salpingo-oophorectomy + peritoneal biopsy + omental biopsy	For the group of malign pathologies: Age, mean ± (range): 59.4 ± 7.99 (44–77) y BMI, mean ± (range): 41.5 ± 9.71 (20.6–56) kg/m^2^	The group of malign pathologies: Operative time: 88.9 (30–245) min Hb change: 0.73 (1–1.8) g/dL No conversion required Pain scores at 6 h,12 h and 24 h: 3.3, 1.76 and 1.03 respectively Blood transfusion: *n* = 1 Bladder injury: *n* = 1 Hospital stay: 1 day No vaginal infections All patients were satisfied with the cosmetic result
Huang L. et al., 2022 [48]	Total: *n* = 1147 Oncological indications: *n* = 14 EC (*n* = 9), CaCx (*n* = 4), OC (*n* = 1)	EC: hysterectomy + BSO + SLND using ICG CaCx: extra facial hysterectomy OC: hysterectomy + BSO + pelvic lymphadenectomy + omentectomy + peritoneal biopsy	Group of oncological indication: EC: Age, mean ± SD: 49.22 ± 4.89 y BMI, mean ± SD: 24.72 ± 2.92 kg/m^2^ CaCx: Age, mean ± SD: 49.50 ± 12.37 y BMI, mean ± SD: 22.80 ± 4.49 kg/m^2^ OC: Age, mean ± SD: 46 y BMI, mean ± SD: 21.10 kg/m^2^	Group of oncological indication: FU: 14 months: No occurrence Operative time: EC: 159.13 ± 37.67 min CaCx: 101.25 ± 26.58 min OC: 340 min EBL: EC: 188.89 ± 188.38 mL CaCx: 50 + /−35.59 mL OC: 200 mL Hospital stay: EC: 6 ±1.41 days CaCx: 5.25 ± 0.96 days OC: 20 days Pain scores at 12 h, 24 h: EC: 3.11 ± 0.6/2.89 ± 0.33 CaCx: 2.75 ± 0.5/2.25 ± 0.5 OC: 3/3 Compication: *n* = 1 for EC group
Lee C.-L. et al., 2022 [49]	Total: *n* = 15 EC st I, grade 1–2: *n* = 15	Hysterectomy + BSO + SLND/PLND	Age, mean ± SD: 52.8 ± 6.8 y BMI, mean ± SD: 27.8 ± 6.4 kg/m^2^	Operative time: 231.4 ± 41.0 min EBL: 122 ± 104.4 mL No blood transfusion Hospital stays: 3.1 ± 1.5 days Only vNOTES: *n* = 11 vNOTES + OUP: *n* = 3 Conversion: *n* = 1 SLN with ICG: *n* = 12 PLND: *n* = 3 FU: no recurrences during 28.6 ± 21.9 months
Huber D. et al., 2022 [50]	Total: *n* = 7 Oncological indications: *n* = 4 EC (*n* = 1), CAH (*n* = 3)	Hysterectomy + BSO + retroperitoneal PSLNB	Age, median (range): 68 (45–83) y BMI, median (range): 26.4 (22.3–44.6) kg/m^2^	Operative time: 113 (81–211) min EBL: 20 (20–400) mL No intraoperative complication No blood transfusion Conversions to CL: *n* = 2 Hospital stay: 2 (2–4) days Postoperative complication: *n* = 1 ^p^
Comba C. et al., 2022 [51]	Total: *n* = 3 EC: *n* = 3	Hysterectomy + SLND	NR	No complication Hospital stay: 1 day EBL < 50 mL
Mat E. et al., 2021 [52]	Total: *n* = 7 Ascites of unknown cause: *n* = 7	Peritoneal biopsy + omental biopsy + US/USO	Age, mean (range): 53.8 (33–66) y BMI, mean (range): 31.7 (25–39) kg/m^2^	Operating time: 47.3 (40–60) min EBL: 5.7 (4–8) mL Hospital stay: 1–2 days Pain scores at 6 h and 24 h: 3.4 and 0.5 respectively No conversion to CL or laparotomy No intraoperative or postoperative complication
Mat E. et al., 2021 [53]	Total: *n* = 6 EC, early stage: *n* = 6	Hysterectomy + BSO	Age, mean ± SD: 53.8 ± 7.5 y BMI, mean (range): 51.4 ± 6.13 (45.6–58.6) kg/m^2^	Operating time: 223.3 ± 25.6 min EBL: minimal No transfusion The Hb level decrease: 1.48 ± 0.17 g/dL on day 1 Hospital stay: 2 days Pain scores at 6 h and 24 h: 3.16 and 0.33 respectively No adjuvant therapy was required
Lowenstein L. et al., 2020 [54]	Total: *n* = 5 Suspicious early-stage OC: *n* = 5	Hysterectomy + BSO + omentectomy ± appendectomy	Age, median (range): 61 (50–72) y BMI, median (range): 27 (23–33) kg/m^2^	Operative time: 103 (92–121) min Omentectomy time: 45 (39–52) min EBL: 150 (20–200) mL Hospital stay: 2 (1–3) days Pain scores at 24 h: 2 (1–2) Demand of paracetamol/patient per 24 h: 3 (2–5) No conversions No complications
Karkia R. et al., 2019 [55]	Total: *n* = 33 Oncological indications: *n* = 1 EC, st I (*n* = 1)	Hysterectomy ± adnexectomy	Total study population: Age, mean (range): 50 (35–75) y BMI, mean (range): 30 (20–53) kg/m^2^ ASA grade, mean (range): 2 (1–3)	Total population study: Operating time: 68.5 (43.0–110.0) min EBL: 269 (50.0–1200.0) mL Hospital of stay: 1.4 (1.0–2.0) nights No conversion to laparotomy or CL No complications No blood transfusion Pain scores at 6 h and discharge were 0
Tantitamit T., 2019 [56]	Total: *n* = 4 EC, st I: *n* = 4	Hysterectomy + BSO + SLND with ICG ^q^	Age, mean ± SD: 60.3 ± 10.2 y BMI, mean ± SD: 25.6 ± 3.29 kg/m^2^	Operative time: 182.75 ± 34.5 min EBL: 67.5 ± 39.4 mL The Hb level decrease: 0.57 ± 0.2 g/dL No intraoperative blood transfusion Hospital stay: 3–5 days No complications No conversions to CL or laparotomy The median number of SLNs: 8.5 (5–16) The overall and bilateral detection rate of SNLs: 100%
Kaya C. et al., 2018 [57]	Total: *n* = 12 Oncological indications: *n* = 1 EC (*n* = 1)	Hysterectomy + BSO	Age: NR BMI: 32.8 kg/m^2^	Operative time: 42 min EBL: 100 mL No complications No pain in the postoperative PE
Lee C.-L. et al., 2014 [58]	Total: *n* = 3 EC, early stage: *n* = 3	Hysterectomy + BSO + BPLND	Age, mean ± SD: 46.3 ± 2.5 y BMI: 27.7 ± 2.4 kg/m^2^	Operative time: 249.3 ± 49.3 min EBL: minimal No intraoperative blood transfusion The Hb decrease on 1st postoperative day: 1.5 ± 0.2 g/dL Hospital stay: 5 (4–5) days The average lymph node yield: 9
Zhang C. et al., 2025 [59]	Ovarian sex cord-stromal tumor, a granulosa cell tumor	RSP-vNOTES hysterectomy + BSO + omentectomy ^r^	Age: 45 y BMI: NR	Operative time: 130 min EBL: 50 mL No analgesics were needed Hospital stay: 3 days
Baekelandt J. et al., 2024 [60]	CaCx adenocarcinoma	Radical hysterectomy + BSO + SLND	Age: 57 y BMI: NR	∙ NR
Can B. et al., 2024 [61]	Endometrioid type Grade 3 EC	SPEL + vNOTES approach: Hysterectomy + BSO + retroperitoneal pelvic + para-aortic lymphadenectomy	Age: 53 y BMI: NR	Operative time: 210 min EBL: 150 mL Pain scores at 6 h and 24 h: 6 and 1 respectively Discharged 30 h after operation
Ng W. et al., 2024 [62]	Total: *n* = 2 Oncological indications: *n* = 1 EC, endometrioid, grade 1 (*n* = 1) ^s^	Hysterectomy + BSO + peritoneal washings	Age: 47 y BMI: 60.4 kg/m^2^	EBL: 150 mL Pain well controlled Discharge postoperative day 1
Erkilinc S. et al., 2024 [63]	EC, endometrioid, grade 1: *n* = 1	SNLD + hysterectomy + salpingo- oophorectomy	Age: 61 y BMI: NR	No complications Discharged postoperative day 2
Guevara R. et al., 2024 [64]	EC, endometrioid, grade 1: *n* = 1	Hysterectomy + double adnexectomy	Age: 81 y BMI: 41.62 kg/m^2^	No postoperative complications Discharged postoperative day 2
Couso A. et al., 2024 [65]	BOT: *n* = 1	Hysterectomy + infracolic omentectomy + appendicectomy	Age: 59 y BMI: NR	Operative time: 115 min No complications Hospital stay: 1 day
Baekelandt J. et al., 2023 [66]	CaCx, superficially invasive squamous cell: *n* = 1	Radical hysterectomy + left side adnexectomy + right side ovario suspension + bilateral parametrium resection + SN resection	Age: 48 y BMI: NR	Discharged at day 3 Complicated by a small right-sided uterovaginal fistula
Kita M. et al., 2023 [67]	Genital tumor with possible clear cell carcinoma on biopsy: *n* = 1	Cervical tumor resection + laparoscopy	Age: 12 y BMI: NR	Operative time: 123 min EBL: minimal R0 resection No postoperative complication FU: no recurrence in 2 y
Li Y. et al., 2022 [68]	EC, low grade endometrioid: *n* = 1	Hysterectomy + BSO + SLND via gasless vNOTES	Age: 43 y BMI: NR	Operative time: 147 min EBL: 50 mL Discharged postoperative day 5 FU: no recurrence or metastasis in 1 y
Hurni Y. et al., 2022 [69]	CaCx, early-stage: *n* = 1	BSLNB through a retroperitoneal vNOTES, after conization	Age: 35 y BMI: NR	Operative time: 96 min No complications 2 pelvic SLNs on the left and 1 on the right side
Mathey M.-P. et al., 2022 [70]	EC, endometrioid, grade 1: *n* = 1	Hysterectomy + BSO + retroperitoneal SLNB with ICG.	Age: 64 y Non obese	Left side 2 SLN, right side 1 SLN Operative time: 113 min, EBL < 100 mL No complications Discharged postoperative day 2 FU: no complications in 3 months
Hurni Y. et al., 2022 [71]	Total: *n* = 2 Oncological indications: *n* = 2 ^t^ Case 1: LGSOC (*n* = 1), Case 2: suspicious ovarian tumor (*n* = 1)	Case 1: VH + BSO + multiple peritoneal biopsies + respected any suspicious lesions (+ infracolic omentectomy + peritoneal washings.) Case 2: hybrid vNOTES right salpingo-oophorectomy, infracolic omentectomy, peritoneal washing and multiple biopsies.	Case 1: Age: 81 y BMI: 22.7 kg/m^2^ Case 2: Age: 62 y, BMI: 16.9 kg/m^2^	Case 1: Operative time: 131 min EBL: 100 mL Hospital stay: 4 days Diagnosis: LGSOC Case 2: Operative time: 97 min, EBL: 30 mL Hospital stay: 1 day Diagnosis: Benign fibrous cystadenoma
Lim Y.H. et al., 2022 [72]	Endometrial sarcoma: *n* = 1	Extraperitoneal PLND + hysterectomy + BSO + omentectomy	Age: 65 y BMI: 35 kg/m^2^	Operative time: 206 min EBL: 200 mL No complications Discharged postperative day 3
Comba C. et al., 2021 [73]	EC, endometrioid, grade 2: *n* = 1	Hysterectomy + BSO + total retroperitoneal BSLND	Age: 46 y ^u^ BMI: 27.4 kg/m^2^	Operative time: 180 min EBL: 20 mL Hospital stay: 1 day No major complications
Kita M. et al. 2021 [74]	Vaginal recurrence of adult type ovarian granulosa cell tumor: *n* = 1	Tumor resection	Age: 39 y BMI: NR	Operative time: 88 min, EBL: minimal R0 resection No complications FU: no recurrence in 1 y
Ju Y.Y. et al., 2021 [75]	EC, endometrioid, grade 1: *n* = 1	Hysterectomy + SLND with ICG ^v^	Age: 54 y BMI: NR	Discharged on the postoperative day 2 No complications
Badiglian-Filho L., 2020 [76]	Serous BOT: *n* = 1	Cystectomy ^w^	Age: 22 y BMI: 42,4 kg/m^2^	Operative time: 120 min EBL: 200 mL
Oh S.H., 2019 [77]	EC, endometrioid, grade 2, st IA: *n* = 1	Hysterectomy + BSO + pelvic lymphadenectomy	Age: 59 y BMI: NR	20 PLNs were retrieved
Htay W.T., 2019 [78]	EC, endometrioid, grade 1: *n* = 1	Hysterectomy + BSO + PSLND with ICG ^x^	Age: 57 y BMI: 29 kg/m^2^	Operative time 120 min EBL 50 mL
Leblanc E., 2016 [79]	EC, endometrioid, grade 2, st IB: *n* = 1	Hysterectomy + BSO + PSLND with ICG	Age: 85 y BMI: 32 kg/m^2^	Discharged the postoperative day 1
Zorrón R. et al., 2008 [80]	OC, adenocarcinoma with ascites and peritoneal carcinomatosis: *n* = 1	vNOTES for diagnostic cancer staging	Age: 50 y BMI: NR	Operative time: 105 min Hospital stay: 2 days No complications No postoperative analgesia needed

Abbreviations: vNOTES, vaginal Natural Orifice Transluminal Endoscopic Surgery; vs, versus; SPLS, Single-port umbilical laparoscopy; EC, endometrial cancer; USO, unilateral salpingo-oophorectomy; BSO, bilateral salpingo-oophorectomy; SLND, sentinel lymph node dissection; ^a^ SLND: vNOTES 4 (6.9%) vs. SPLS 6 (9.5%). SD, standard deviation; y, years; BMI, body mass index; kg, kilogram; m, meter; h, hours; SFI, Sexual Function Index; ICG, indocyanine green; MB, methylene blue; st, stage; SLN, sentinel lymph node; EBL, estimated blood loss; RS, robotic surgery; NR, not reported; CL, conventional laparoscopy; OC, ovarian cancer; ^b^ Primary staging surgery group: hysterectomy (*n* = 12) ± omentectomy (*n* = 4) ± PLND (*n* = 3), Fertility-sparing surgery group: unilateral salpingo-oophorectomy (*n* = 2)/unilateral cystectomy (*n* = 2) ± omentectomy (*n* = 1), restaging surgery group: total hysterectomy + left salpingo-oophorectomy + omentectomy and IDS group: total hysterectomy + omentectomy ± pelvic peritonectomy. IDS, interval debulking surgery; mL, milliliter; min, minutes; FU, follow-up; EIN, endometrial intraepithelial neoplasia; Hb, hemoglobin; ICU, Intensive care unit; ML, multiport laparoscopy; PSDR, the detection rate of a pelvic LN on at least one side; GR-vNOTES, gasless robot-assisted transvaginal natural orifice transluminal endoscopic surgery; TR-vNOTES, traditional robot-assisted transvaginal natural orifice transluminal endoscopic surgery; ^c^ Patients with early cervical cancer. ^d^ 5 parients with early cervical cancer and one with early endometrial carcinoma. ^f^ 1 patient with early endometrial carcinoma in TR-vNOTES. ^g^ Unilateral or bilateral. ^h^ Conversion to conventional laparoscopy because of failed mapping on both sides of the pelvis. LGSOC, low-grade ovarian serous carcinoma; CaCx, cervical cancer; BOT, borderline ovarian cancer; PSLND, pelvic sentinel lymph node dissection; US, unilateral salpingectomy; BS, bilateral salpingectomy; ^i^ Except one case of ovarian spillage. ^j^ Included one surgical infection (9.1%) and two postoperative cystitis (18.2%). CIN, Cervical intraepithelial neoplasia; ^k^ On average, three nodes were resected per case. NR, not reported; ^l^ Conversions to laparotomy. ^m^ Preoperatively, endometrial cancer was the only known indication for surgery. However, postoperative histopathological analysis revealed additional diagnoses, including ovarian adult granulosa cell tumor, ovarian mucinous borderline tumor, and high-grade serous ovarian carcinoma. BPSLND, bilateral pelvic sentinel lymph node dissection; SAM, suspicious adnexal masses; ^n^ In one case hybrid approach of vNOTES and transubilical trocar for a 17 cm ovarian lesion performed. BSLND, bilateral sentinel lymph node dissection; ^o^ Directly attributable to the SLN dissection, 1 patient had a transient adductor paresis that resolved within three days and 1 patient had transection of an obturator nerve without sequelae. PLND, pelvic lymph node dissection; OUP, one umbilical port; CAH, complex atypical hyperplasia; ^p^ 1 patient developed deep venous thrombosis on the 20th postoperative day and later asymptomatic vaginal vault hematoma; ^q^ One of the four patients was operated on at another hospital. PE, pelvic exam; BPLND, bilateral pelvic lymph node dissection; RSP-vNOTES, robotic single port- vaginal Natural Orifice Transluminal Endoscopic Surgery; ^r^ The initial intervention involved a RSP-vNOTES left oophorectomy, whereas the subsequent procedure, performed following the final pathological diagnosis, consisted of RSP-vNOTES hysterectomy, adnexectomy, and omentectomy. SPEL, single port extraperitoneal laparoscopy; ^s^ Case 1 was a suspicious ovarian torsion. SN, sentinel node; ^t^ suspicious ovarian tumors ^u^ The patient previously underwent a right hemicolectomy via a midline incision for colon adenocarcinoma in 2013, followed by 12 cycles of chemotherapy. ^v^ Performed extraperitoneal sentinel lymph node biopsy by vNOTES. ^w^ Also, performed resection of the left tube, due to firm adhesion to the cyst. PLN, pelvic lymph node; ^x^ The first procedure consisted of vNOTES total hysterectomy with BSO for a myoma indication. The pathological report revealed endometrioid adenocarcinoma Grade 1, and a secondary vNOTES pelvic sentinel lymph node dissection was subsequently performed.

**Table 3:** Quality assessment of observational cohort studies assessing vaginal natural orifice transluminal endoscopic surgery (vNOTES) for gynecological procedures in patients with a gynecological malignancy, according to the Newcastle-Ottawa Scale (NOS).

First Author, Publication Year, Reference	Selection	Comparability	Outcome	NOS Score
Gungorduk K. et al., 2025 [20]	****	**	***	9
Arkan K. et al., 2025 [21]	****	*	***	8
Şimşek E et al., 2025 [22]	****	**	***	9
Fong K.Y. et al., 2025 [23]	***	-	***	6
Mat E. et al., 2024 [24]	****	-	***	7
Comba C. et al., 2024 [25]	****	-	***	7
Deng L. et al., 2023 [26]	****	*	***	8
Bouchez MC. et al., 2023 [27]	***	-	***	6
Mei Y. et al., 2023 [28]	****	*	***	8
Merlier M. et al., 2022 [29]	****	*	**	7
Wang Y. et al., 2021 [30]	****	-	**	6

* = 1 point; ** = 2 points; *** = 3 points; **** = 4 points in each Newcastle-Ottawa (NOS) domain (Selection, max 4; Comparability, max 2; Outcome, max 3). Total NOS score is the sum of domain points.

### 3.2. Case-Series

The outcomes of the eligible case series included in this systematic review are summarized in Table 2. In total, 28 case series were identified, comprising 16 retrospective, 4 prospective and 6 combined prospective and retrospective studies, while the study design was unspecified in 2 studies. These studies included 3107 patients who underwent vNOTES surgery for gynecologic indications. Among these, 592 (19.05%) patients underwent procedures for confirmed or suspected gynecologic malignancies or endometrial hyperplasia.

These studies were conducted between 2014 and 2025 across tertiary care centers in Switzerland, Turkey, the United States, Singapore, Brazil, Belgium, Spain, Taiwan, Israel, the United Kingdom and China. Patient demographics varied across studies. The reported mean patient age ranged from 41 years [35] to 72.5 years [31], with the latter study including exclusively patients aged over 65 years. The median BMI in the vNOTES case series ranged from 25.6 kg/m^2^ [36,56] to 51.4 kg/m^2^ [53]. Notably, three studies specifically enrolled obese or morbidly obese patients who underwent vNOTES procedures [42,52,53].

More specifically, 534 (90.2%) patients were diagnosed with endometrial carcinoma [31,32,33,37,38,39,40,41,42,43,44,46,49,50,51,56,58], including 54 patients described as having “early-stage endometrial/cervical malignancy or premalignancy,” without further specification [41]. One (0.16%) patient was diagnosed with uterine carcinosarcoma [31], 28 (4.72%) with ovarian or tubal neoplasia [31,34,44,48,54], and 5 (0.84%) with borderline ovarian tumors [31,34]. Cervical intraepithelial neoplasia or low-grade cervical cancer was reported in 13 (2.19%) patients, while 10 (1.68%) patients were explicitly diagnosed with cervical cancer [31,41,46]. Furthermore, 11 (1.85%) patients presented with ascites, including cases of unknown cause (*n* = 7), peritoneal carcinomatosis (*n* = 3), and gastric carcinoma (*n* = 1) [47,52].

The predominant oncologic procedure performed via the vNOTES approach was total hysterectomy, most commonly accompanied by bilateral or unilateral salpingo-oophorectomy. For staging purposes, the procedure was extended to include SLN mapping in 347 (58.61%) cases of endometrial and cervical cancer [31,32,33,37,38,39,40,41,42,43,44,46,48,49,50,51,56,58], while pelvic lymph node dissection was performed in three (0.5%) patients with endometrial cancer [49]. Across these staging procedures, the mean number of lymph nodes excised ranged from 9 to 12.5 per patient [39,58]. Additional oncologic staging procedures, including infracolic omentectomy (*n* = 51, 8.61%) [31,34,43,44,47,48,54] and peritoneal biopsies (*n* = 10, 1.68%) for ovarian cancer or ascites of unknown origin [48,52], were also successfully performed via the vNOTES approach. Moreover, concomitant appendectomy was reported in four (0.67%) patients [43,44,54]. Notably, in one (0.16%) case of cervical cancer, the planned total hysterectomy was aborted following the intraoperative identification of SLN metastasis [41].

Of note, Yang et al. reported the application of a robotic-assisted adaptation of the technique, referred to as Robot-Assisted Vaginal Natural Orifice Transluminal Endoscopic Surgery (RA-vNOTES), in a cohort of 292 patients, including two (0.33%) patients diagnosed with endometrial cancer [35].

Regarding the clinical outcomes, the reported mean EBL ranged from 43.6 mL [45] to 269 mL [55], indicating low intraoperative blood loss across most procedures. A single case requiring reintervention due to significant postoperative bleeding was described by Baekeland et al. [38]. Mean operative time varied widely across the included studies, ranging from 68.5 [55] to 340 min [48]. The maximum duration of 340 min, reported by Huang et al. [48], was attributed to the inclusion of patients undergoing vNOTES for ovarian cancer. Conversion to conventional laparoscopy was necessary in eight cases due to bleeding and bladder injury [31,38,40,50,58].

Furthermore, the maximum postoperative visual analog scale (VAS) pain score recorded at 24 h was 3/10 [44]. Across the included case series, the duration of hospital stay following vNOTES ranged from same-day [35,37,41,47,51] discharge to 20 [48] days, with 20 studies reporting mean or median durations of 1 to 3 days regardless of surgical complexity [31,32,33,35,36,37,38,39,41,42,43,44,45,47,50,51,52,53,54,55]. Intraoperative complications included thermal injury to the colon [36] and bladder injury [47], whereas postoperative complications included cystitis, deep vein thrombosis, surgical site infection, pelvic hematoma, and transient adductor muscle paresis.

Table 4 presents the quality assessment of the included studies according to the NIH Quality Assessment Tool. 14 studies were rated as good quality, 13 as fair, and one as poor quality.

### 3.3. Case Reports

Table 2 also includes the surgical outcomes of the 22 case reports retrieved through the systematic literature review. These studies encompassed a total of 23 patients, with the case report by Hurni et al. contributing two cases of suspicious ovarian tumors [71].

The vNOTES procedures performed for confirmed or suspected malignancies were conducted in tertiary care centers located in Belgium, Turkey, Singapore, China, Spain, Japan, Switzerland, Brazil, the Republic of Korea, Taiwan, and France. While the patient population primarily consisted of adults aged 22 [76] to 85 years [79], one pediatric case involving a 12-year-old patient with a clear cell cervical tumor was also reported [67]. BMI values varied considerably across studies, ranging from 16.9 kg/m^2^ [71] to 60.4 kg/m^2^ [62].

The majority of patients, ten (43.47%) in total, underwent vNOTES procedures for endometrial carcinoma [61,62,63,64,68,70,73,75,77,78,79], while four (17.39%) patients were diagnosed with cervical cancer [60,66,67,69]. Additionally, four (17.39%) patients were diagnosed with ovarian malignancy [59,71,80], and two (8.69%) patients were managed for borderline ovarian tumors [65,76]. Furthermore, one (4.34%) patient was operated on for endometrial sarcoma [72], and another (4.34%) for vaginal tumor recurrence of an adult-type ovarian granulosa cell tumor [74].

The surgical intervention that was most commonly performed was total hysterectomy, combined with either unilateral or bilateral salpingo-oophorectomy, with or without pelvic and/or para-aortic lymphadenectomy or SLN mapping. Couso et al. performed a vNOTES total hysterectomy in conjunction with infracolic omentectomy and appendectomy for the management of a borderline ovarian tumor [65]. Additionally, Zhang et al. performed Robotic single port (RSP)–vNOTES hysterectomy and bilateral salpingo-oophorectomy with omentectomy for staging following resection of an ovarian granulosa-cell tumor [59]. Similarly, Hurni et al. supplemented the standard procedure with infracolic omentectomy and peritoneal biopsies in patients with suspected ovarian malignancies [69]. Also, Zorrón et al. performed diagnostic cancer staging surgery for peritoneal carcinomatosis [80], and Kita et al. achieved tumor resection of a vaginal recurrence via vNOTES [74].

Operative time for vNOTES hysterectomy varied across the case reports, ranging from 113 min [70] to 210 min [61]. Hurni et al. reported an operative time of 96 min for bilateral sentinel lymph node biopsy performed via a retroperitoneal vNOTES approach, after positive conization [69]. In another case, Kita et al. described tumor resection via vNOTES in 88 min for a recurrent mass located in the patient’s left vaginal wall, occurring 23 years after initial surgery for an ovarian granulosa cell tumor [74].

EBL was consistently low across all reported cases, ranging from 20 mL [73] to 200 mL [74,76]. No intraoperative or postoperative complications were observed. The length of hospital stay varied among patients, with discharge occurring between postoperative days 1 and 5. Ultimately, no cases of recurrence were reported during the follow-up period, which ranged between 1 and 2 years.

Table 5 summarizes the JBI-based quality assessment of the included case reports.

## 4. Discussion

This study aimed to assess the feasibility, potential benefits, and limitations of the vNOTES approach for gynecologic malignancies based on a systematic review of the current literature. A total of 61 studies were included, comprising 11 cohort studies, 28 case series, and 22 case reports, encompassing a pooled sample of 926 patients diagnosed with suspected or confirmed gynecologic malignancies who were treated using the vNOTES approach. The most frequently performed procedure was total hysterectomy combined with bilateral salpingo-oophorectomy and sentinel lymph node biopsy, with early-stage endometrial cancer being the predominant oncological indication. The clinical and perioperative outcomes assessed in the included studies encompassed estimated blood loss, operative time, sentinel lymph node detection rate, conversion rates, postoperative pain, hospital length of stay, and complications.

In recent years, the vNOTES approach has gained increasing recognition, particularly regarding its safety and efficacy in the management of benign gynecological conditions [81,82,83,84]. Additionally, current evidence comparing vNOTES with other MIS approaches for benign gynecological surgeries shows significant advantages in operative time, length of hospitalization, and postoperative pain levels [85,86,87,88,89]. Notably, a recent meta-analysis by Michener et al. highlighted that vNOTES offers lower pain scores and comparable complication rates relative to conventional laparoscopy [90]. Moreover, the safety and applicability of vNOTES have been evaluated in specific patient populations. Nef et al. reported its feasibility and safety in elderly patients [31]. Similarly, Burnett et al. established that vNOTES was a viable option for obese patients, noting its association with accelerated recovery; thereby enabling the majority of patients to be managed in an outpatient setting without the need for readmission [42].

Τhe role of vNOTES in the management of gynecologic malignancies has attracted increasing scientific interest, and its use may represent a feasible alternative surgical approach [91]. The majority of published studies to date have primarily focused on its use in the surgical staging of early endometrial cancer, with particular emphasis on the feasibility and outcomes of sentinel lymph node biopsy via this approach [92,93]. Baekelandt et al. described a novel retroperitoneal vNOTES approach, enabling transvaginal access to the pelvic and paraaortic retroperitoneal spaces for the performance of sentinel lymph node biopsy, while Huber et al. demonstrated that the implementation of this technique is feasible and safe [50,94]. Furthermore, Deng et al. compared vNOTES with conventional laparoscopy and showed that it was not inferior in sentinel lymph node detection for endometrial cancer [24]. A key benefit of the transvaginal approach lies in its caudal-to-cranial direction of lymph node dissection, which reflects the physiological lymphatic flow from the uterus. This anatomical alignment reduces the likelihood of misidentifying and excising a secondary lymph node rather than the true sentinel node. Additionally, the shorter anatomical distance to the SLN via the vaginal route contributes to reduced operative time and surgical morbidity by minimizing the extent of surgical dissection. Notably, the retroperitoneal access utilized for SLN mapping avoids the manipulation of the peritoneal cavity, thereby preventing the formation of postoperative peritoneal adhesions [38].

Furthermore, for obese or elderly patient populations, where the prevalence of endometrial cancer is higher, vNOTES provides substantial benefits over conventional laparoscopy. Specifically, it obviates the need for steep Trendelenburg positioning and high-pressure pneumoperitoneum required during conventional laparoscopy, thereby reducing the cardiopulmonary strain typically induced by these factors [29,43,53,95]. Particularly, in obese patients, the placement and manipulation of instruments are facilitated, as the vNOTES technique circumvents the challenges imposed by excessive abdominal wall thickness. Moreover, increased abdominal adiposity can impede laparoscopic colpotomy and vaginal cuff closure, technical challenges that are effectively resolved by the direct access afforded by the transvaginal approach, ultimately resulting in shorter operative times [42,95,96].

The management of early-stage cervical cancer is closely contingent upon the potential lymphatic spread of the disease, which can be assessed through sentinel lymph node biopsy [97,98]. In the absence of lymph node involvement, radical surgery with extended lymphadenectomy is warranted, whereas the presence of metastatic lymph nodes indicates the need for chemoradiotherapy combined with brachytherapy [97]. The retroperitoneal vNOTES approach described by Baekelandt et al. offers full and straightforward access to the retroperitoneal space for sentinel lymph node identification without the need for concurrent hysterectomy. Furthermore, by avoiding entry into the peritoneal cavity, it reduces the risk of adhesion formation and mitigates adverse complications related to subsequent radiotherapy in cases of positive lymph nodes. Moreover, due to its association with reduced postoperative pain and faster recovery compared to other surgical approaches, this technique facilitates earlier initiation of adjuvant therapy, when clinically indicated [28]. Therefore, this approach represents a pivotal modality in the armamentarium of gynecologic oncologists for the two-step strategy in managing early cervical cancer, facilitating definitive pathological assessment of the sentinel lymph node while minimizing morbidity. Hurni et al. advocate its use in patients with negative conization margins and low-risk features for parametrial involvement, aiming to reduce the risk of tumor cell dissemination associated with surgical manipulation [69].

The application of the vNOTES approach in ovarian cancer treatment remains uncertain, primarily because of the scarcity of supporting studies. In their respective studies, Lowenstein et al. (5 cases) and Hurni et al. (18 cases) demonstrated the feasibility of staging early-stage ovarian cancer using this technique [44,54]. The primary benefit of utilizing the vNOTES technique in this patient population lies in the accelerated recovery attributed to the absence of abdominal incisions and associated nerve injury, thereby facilitating the prompt initiation of adjuvant chemo-radiotherapy. However, its use raises concerns regarding potential understaging due to restricted visualization of certain anatomical structures, as well as the risk of tumor cell dissemination and subsequent upstaging in the event of accidental rupture of the mass. Such limitations may be mitigated by employing articulating instruments and variable-view rigid endoscopes, and by ensuring that specimen extraction is conducted entirely within dedicated endobags to prevent tumor cell dissemination [99,100].

Despite its clear benefits, the vNOTES technique may present certain challenges, such as instrument crowding due to insertion through a single narrow port, which restricts the surgeon’s range of motion. Moreover, the surgeon must be highly proficient in vaginal access and maintain spatial orientation despite the distinctive visual perspective, necessitating a dedicated learning curve [39]. A significant limitation of the vNOTES approach is the requirement for a patent pouch of Douglas, which must be free of adhesions or space-occupying masses to ensure safe entry into the peritoneal cavity. Similarly, the absence of pelvic organ prolapse can compromise surgical exposure, rendering vaginal access more technically demanding and posing a significant challenge for the surgeon. Lastly, the introduction of instruments through a non-sterile natural orifice poses a theoretical risk of ascending infection and peritoneal contamination, with potentially severe clinical implications for the inherently immunocompromised oncological population.

Several limitations must be considered. The risk of publication and selection bias is significant, as the current systematic review is largely based on small case series, with a limited number of comparative studies involving control groups. Case reports were included to comprehensively capture the available literature, although they provide a lower tier of evidence for establishing robust conclusions. Furthermore, a formal meta-analysis or subgroup statistical processing was not feasible due to the significant heterogeneity in study design, gynecological cancer types, patient characteristics, and surgical indications; this variability could limit the external validity and generalizability of the accumulated results. Additionally, the utilization of vNOTES in gynecological oncology is currently restricted to a modest number of reported cases worldwide, predominantly involving patients with early-stage disease, with the relatively short follow-up duration precluding drawing definitive oncological conclusions. Consequently, the available data in the literature concerning these patients are derived from specific centers and countries. Moreover, in instances where study periods overlap, the duplication of cases across different reports cannot be entirely ruled out.

Future investigations should focus on well-designed, multicenter randomized controlled trials comparing vNOTES with conventional MIS approaches, with an emphasis on long-term outcomes such as postoperative recovery, recurrence rates, and quality of life. Additionally, comparative studies are required to evaluate vNOTES against robotic or laparoscopic surgery in patients with gynecological malignancies and specific clinical characteristics, such as the obese and elderly. Moreover, comparative studies evaluating the clinical outcomes of vNOTES specifically between obese and non-obese patient cohorts would provide valuable insights into its distinct advantages. It is also imperative to evaluate the efficacy and safety of the vNOTES approach compared with other surgical modalities in patients who have undergone neoadjuvant chemotherapy, a clinical setting where optimized perioperative care is critical. Finally, high-quality evidence is needed to further elucidate patient-reported outcomes, cost-effectiveness, and the learning curve associated with this technique.

## 5. Conclusions

Based on the accumulated data, the application of the vNOTES approach appeared to be feasible and at least non-inferior to standard surgical modalities for patients with gynecological malignancies, particularly those with early-stage disease. However, it should be acknowledged that the available data are derived from heterogeneous studies with relatively small patient samples, thereby limiting the ability to draw definitive conclusions. Future research should focus on large-scale, multicenter randomized controlled trials to validate these findings and to further define the role of this surgical approach in clinical practice.

## Figures and Tables

**Table 4:** Quality assessment of observational case series studies assessing vaginal natural orifice transluminal endoscopic surgery (vNOTES) for gynecological procedures in patients with a gynecological malignancy, according to the National Institute Health (NIH) Quality Assessment Tool for Case Series Studies.

First Author, Publication Year, Reference	Yes	No	Other (CD, NR, NA) *	Quality Rating (Good, Fair, or Poor)
Nef J. et al., 2025 [31]	6	1	2	Fair
Gungorduk K. et al., 2025 [32]	6	1	2	Fair
Hanedan C. et al., 2025 [33]	7	1	1	Good
Kellerhals G. et al., 2025 [34]	7	0	2	Good
Yang Q. et al., 2025 [35]	5	2	2	Fair
Tan R.C.A. et al., 2025 [36]	9	0	0	Good
Simsek E et al., 2024 [37]	5	2	2	Fair
Baekelandt J. et al., 2024 [38]	7	0	2	Good
Matak L. et al., 2024 [39]	7	1	1	Good
Huber D. et al., 2024 [40]	6	2	1	Fair
Zarragoitia J. et al., 2024 [41]	4	3	2	Fair
Burnett AF. et al., 2024 [42]	6	2	1	Fair
Hurni Y. et al., 2023 [43]	8	0	1	Good
Hurni Y et al., 2023 [44]	8	0	1	Good
Mat E. et al., 2023 [45]	6	2	1	Fair
Burnett AF. et al., 2023 [46]	5	2	2	Fair
Kale A. et al., 2022 [47]	7	1	1	Good
Huang L. et al., 2022 [48]	6	3	0	Fair
Lee CL. et al., 2022 [49]	8	0	1	Good
Huber D. et al., 2022 [50]	8	0	1	Good
Comba C. et al., 2022 [51]	2	5	2	Poor
Mat E. et al., 2021 [52]	7	1	1	Good
Mat E. et al., 2021 [53]	7	1	1	Good
Lowenstein L. et al., 2020 [54]	6	1	2	Fair
Karkia R. et al., 2019 [55]	8	1	0	Good
Tantitamit T. et al., 2019 [56]	6	1	2	Fair
Kaya C. et al., 2018 [57]	7	1	1	Good
Lee CL. et al., 2014 [58]	6	2	1	Fair

* CD: cannot determine, NA: not applicable, NR: not reported.

**Table 5:** Quality assessment of case report studies assessing vaginal natural orifice transluminal endoscopic surgery (vNOTES) for gynecological procedures in patients with a gynecological malignancy, according to the Joanna Briggs Institute (JBI) Critical Appraisal Checklist for Case Reports.

First Author, Year of Publication, Reference	Yes	No	Unclear	Not Applicable
Zhang C. et al., 2025 [59]	8	-	-	-
Baekelandt J. et al., 2024 [60]	3	3	2	-
Can B. et al., 2024 [61]	7	1	-	-
Ng W. et al., 2024 [62]	4	2	2	-
Erkilinc S. et al., 2024 [63]	8	-	-	-
Guevara R. et al., 2024 [64]	7	-	1	-
Couso A. et al., 2024 [65]	7	1	-	-
Baekelandt J. et al., 2023 [66]	8	-	-	-
Kita M. et al., 2023 [67]	8	-	-	-
Li Y. et al., 2022 [68]	8	-	-	-
Hurni Y. et al., 2022 [69]	8	-	-	-
Mathey MP et al., 2022 [70]	8	-	-	-
Hurni Y. et al., 2022 [71]	8	-	-	-
Lim Y.H. et al., 2022 [72]	7	1	-	-
Comba C. et al., 2021 [73]	7	1	-	-
Kita M. et al., 2021 [74]	8	-	-	-
Ju Y.Y. et al., 2021 [75]	7	-	1	-
Badiglian-Filho L. et al., 2020 [76]	7	1	-	-
Oh SH et al., 2019 [77]	7	1	-	-
Htay WT et al., 2019 [78]	7	1	-	-
Leblanc E. et al., 2016 [79]	6	2	-	-
Zorron R. et al., 2008 [80]	8	-	-	-

## Data Availability

The data used in this study are available upon request.

## Supplementary Materials

The following supporting information can be downloaded at https://www.mdpi.com/article/10.3390/jcm15114089/s1, Figure S1a: Preferred Reporting Items for Systematic Reviews and Meta-Analyses (PRISMA) 2020 Checklist; Figure S1b: Preferred Reporting Items for Systematic Reviews and Meta-Analyses (PRISMA) 2020 Abstract Checklist.

## Abbreviations

The following abbreviations are used in this manuscript:

GLOBOCAN	Global Cancer Observatory
vNOTES	Vaginal Natural Orifice Transluminal Endoscopic Surgery
MeSH	Medical Subject Headings
PRISMA	Preferred Reporting Items for Systematic Reviews and Meta-Analysis
NOS	Newcastle–Ottawa Scale
ΝΙH	National Institute of Health
JBI	Joanna Briggs Institute
vNOTES-H	Vaginal Natural Orifice Transluminal Endoscopic Surgery-Hysterectomy
VH	vaginal hysterectomy
BMI	median body mass index
SLND	sentinel lymph node dissection
PLND	pelvic lymph node dissection
MIS	minimally invasive surgery
SPLS	single-port laparoscopy
CL	conventional laparoscopy
ML	multiport laparoscopy
EBL	estimated blood loss
SLN	sentinel lymph node
ICG	indocyanine green
RA-vNOTES	Robot-Assisted Vaginal Natural Orifice Transluminal Endoscopic Surgery
VAS	visual analog score
RSP	Robotic single port
